# Anesthetic management of living-donor lung transplantation for end-stage COVID-19 lung failure

**DOI:** 10.1186/s40981-022-00512-9

**Published:** 2022-03-15

**Authors:** Satoshi Shimizu, Kayo Kimura

**Affiliations:** Department of Anesthesia, Kyoto University Hospital, 54 Shogoin-kawahara-cho, Sakyo-Ku, Kyoto City, Kyoto Prefecture Japan

**To the editor:**

The coronavirus disease 2019 (COVID-19) pandemic remains ongoing [1]. Lung transplantation is a potentially life-saving treatment for end-stage post-COVID-19 lung failure [2–4]. Few brain-dead donor organs are available in Japan; therefore, living-donor lung transplantation (LDLT) plays a vital role in such cases. We report the first case of anesthetic management of LDLT for post-COVID-19 respiratory failure.

A 57-year-old woman was on veno-venous extracorporeal membrane oxygenation (VV-ECMO) due to severe post-COVID-19 lung injury, even after systemic inflammation subsided. Three months after disease onset, she was transferred to our institution for LDLT, as she recovered to the point where she could actively engage in rehabilitation. She had a Glasgow Coma Scale score of E4VTM6, blood pressure of 105/69 mmHg, and a sinus heart rate of 80/min. The patient breathed spontaneously through a tracheostomy. Gaseous exchange was completely dependent on VV-ECMO, and her oxygen saturation (SpO_2_) was 93% under room air. She had a short neck with limited mobility and her mouth opened to only about 1.5 horizontal fingers. Blood examination revealed anemia, thrombocytopenia, decreased fibrinogen levels, and increased D-dimer levels, presumably due to prolonged ECMO use. The activated partial thromboplastin time (APTT) was prolonged on 14,400–16,320 units/day of heparinization (Table 1). She had unimpaired biventricular function with no valvular disease.

**Table 1:** Blood examination on transfer to our institution

**Blood count and coagulation test results**	
White blood cells	7.79×10^3^/μL
Red blood cells	296×10^4^/μL
Hemoglobin	9.6 g/dL
Hematocrit	28 %
Platelet count	6.5×10^4^/μL
Prothrombin time	1.11 (international normalized ratio (INR))
Activated partial thromboplastin time	62.5 s
Fibrinogen	66 mg/dL
D-dimer	18.7 μg/mL
**Arterial blood gas analysis (room air)**	
pH	7.469
PaCO_2_	33.9 mmHg
PaO_2_	65.8 mmHg
HCO_3_^−^	24.1mmol/L
BE	0.4 mmol/L
SaO_2_	93.5%

VV-ECMO at 2800 rpm, blood flow 3.0 L/min, sweep gas 4 L/min with FiO_2_ 1.0

General anesthesia was induced with 5 mg of midazolam, 250 μg of fentanyl, and 100 mg of rocuronium and maintained with propofol and remifentanil. A 35Fr double-lumen tube was inserted orally using a McGRATH^TM^. An arterial line was placed in the right radial artery. Pulmonary and central venous catheters were inserted into the left internal jugular vein. Although most previous cases reporting dead-donor lung transplantation switched from VV-ECMO to VA-ECMO perioperatively, cardiopulmonary bypass (CPB) was established to avoid the lethal risk of circuit failure on switching due to insufficient evacuation or air suctioning upon venous cannulation into the right atrium. After the right lower lobe from her son and the left from her husband were transplanted, she was weaned from CPB under 4 mcg/kg/min of dobutamine and 0.05 mcg/kg/min of noradrenaline. She did not require ECMO support postoperatively. Anesthesia and operation times were 812 and 657 min, respectively. The estimated blood loss was 12,370 mL and urine output was 1410 mL. Crystalloid and colloid infusions were 1900 mL and 2000 mL, respectively. The patient was transfused with packed red cells (3360 mL), fresh frozen plasma (5040 mL), and platelet concentrates (800 mL). Cefozopran, levofloxacin, and micafungin were administered perioperatively for a week, referring to the serum creatinine level. The heparin was not administered due to concerns about postoperative hemorrhage. She was discharged from the intensive care unit on postoperative day (POD) 26. Although weaning from mechanical ventilation took approximately 2 months, she recovered well and was transferred to a rehabilitation hospital on POD 131. Our case indicates that anesthetic management for LDLT for end-stage COVID-19 lung failure is not essentially different from that indicated for other end-stage lung failures, except that the severe tissue adhesion and coagulopathy specific to COVID-19 would cause massive bleeding.

## Data Availability

Not applicable.

## Abbreviations

COVID-19: Coronavirus disease 2019
LDLT: Living-donor lung transplantation
VV-ECMO: Veno-venous extracorporeal membrane oxygenation
SpO_2_: Oxygen saturation
APTT: Activated partial thromboplastin time
CPB: Cardiopulmonary bypass
